# Alfalfa xenomiR-162 targets G protein subunit gamma 11 to regulate milk protein synthesis in bovine mammary epithelial cells

**DOI:** 10.5713/ab.23.0370

**Published:** 2024-01-20

**Authors:** Guizhi Meng, Hongjuan Duan, Jingying Jia, Baobao Liu, Yun Ma, Xiaoyan Cai

**Affiliations:** 1 Key Laboratory of Ruminant Molecular and Cellular Breeding of Ningxia Hui Autonomous Region, College of Animal Science and Technology, Ningxia University, Yinchuan 750021, China

**Keywords:** Alfalfa xenomiR-162, Bovine Mammary Epithelial Cells, mTOR Pathway, Milk Protein, Proliferation

## Abstract

**Objective:**

It was shown that microRNAs (miRNAs) play an important role in milk protein synthesis. However, the post-transcriptional regulation of casein expression by exogenous miRNA (xeno-miRNAs) in ruminants remains unclear. This study explores the regulatory roles of alfalfa xeno-miR162 on casein synthesis in bovine mammary epithelial cells (bMECs).

**Methods:**

The effects of alfalfa xenomiR-162 and G protein subunit gamma 11 (GNG11) on proliferation and milk protein metabolism of bMECs were detected by 5-Ethynyl-2′-Deoxyuridine (EdU) staining, flow cytometry, cell counting kit-8 (CCK-8), enzyme-linked immunosorbent assay, quantitative real-time polymerase chain reaction (qRT-PCR), and Western blot. Dual-luciferase reporter assay was used to verify the targeting relationship between GNG11 and xenomiR-162.

**Results:**

Results showed that over-expression of xenomiR-162 inhibited cell proliferation but promoted apoptosis, which also up-regulated the expression of several casein coding genes, including CSN1S1, CSN1S2, and CSN3, while decreasing the expression of CSN2. Furthermore, the targeting relationship between GNG11 and xenomiR-162 was determined, and it was confirmed that GNG11 silencing also inhibited cell proliferation but promoted apoptosis and reduced the expression of casein coding genes and genes related to the mammalian target of rapamycin (mTOR) pathway.

**Conclusion:**

Alfalfa xenomiR-162 appears to regulate bMECs proliferation and milk protein synthesis via GNG11 in the mTOR pathway, suggesting that this xeno-miRNA could be harnessed to modulate CSN3 expression in dairy cows, and increase κ-casein contents in milk.

## INTRODUCTION

microRNAs (miRNAs) are endogenous non-coding single-stranded RNAs commonly found in cells of living organisms, which are involved in regulating gene expression during individual development and play “housekeeping” roles by regulating up to 60% of protein-coding genes within a genome [1]. Earlier studies observed cells could secrete miRNAs that enter recipient cells to regulate gene expression in a “cross-border regulation” manner [2]. miR-168a from rice was first found to accumulate in the liver of mice via the digestive tract, where it targeted low density lipoprotein receptor adaptor protein 1 (LDLRAP1), resulting in increased levels of plasma low-density lipoprotein [3]. Other exogenous, plant-derived miRNAs, or so-called xeno-miRNAs, have been subsequently detected in other animals [4], xenomiR-156a, miR-168a, and miR-166a were detected in calf serum in 2012 [3], however, which plant do these xeno-miRNAs come from and what function of them had not been studied in dairy cows.

Alfalfa is widely used as a nutrient-rich forage in livestock feed and is known to improve protein content in dairy milk production. Milk protein is the primary nutritional component of milk and an essential indicator of milk quality. Milk protein is synthesized in bovine mammary epithelial cells (bMECs) and is known to be regulated by several signaling pathways, including the protein kinase B (AKT)-mammalian target of rapamycin (mTOR), Janus tyrosine Kinase-Janus tyrosine Kinase (JAK-STAT) and phosphatidylinositol 3-kinase (PI3K) pathways. Moreover, these pathways may be regulated by endogenous miRNAs, such as miR-221 or miR-142-3p, that can inhibit bMECs proliferation by targeting STAT5a or the prolactin receptor (PRLR), respectively [5,6]. However, studies have investigated the mechanisms by which xeno-miRNAs might regulate genes responsible for breast cancer [7]. In addition, our research group determined that over-expression of alfalfa xeno-miR168b results in inhibition of bMECs proliferation and reduction of lipid droplets and triglyceride content in bMECs [8]. Thus, we hypothesize there is potential for exploiting specific components of alfalfa to improve milk protein.

Dairy milk is generally considered an important protein source in the human diet. The main milk proteins are casein and whey protein; whey protein accounts for 20 percent of the milk protein portion, while casein accounts for 80 percent; casein consists of αs1-casein (CSN1S1), αs2-casein (CSN1S2), β-casein (CSN2), γ-casein and κ-casein (CSN3) [9]. There are essential amino acids in casein, which is the only milk protein that can solidify and form stable microglue particles with calcium and phosphorus to increase the content of calcium and phosphorus in the milk [10]. The results of this study will lay a foundation for clarifying the molecular regulation mechanism of alfalfa cross-border regulation of milk protein synthesis in dairy cows and provide a novel theoretical basis for applying alfalfa-derived miRNAs to improve κ-casein contents in milk production.

## MATERIALS AND METHODS

### Ethical statement

The animal study protocol was approved by the Ningxia University Technology Ethics Committee, Yinchuan, China (protocol code NXU-23-80).

### Serum and whey isolation from Holstein cows

Milk and blood of Holstein cows from Helanshan Dairy Industry Co., Ltd., in Ningxia Nongken, China, were collected and analyzed. Milk samples were collected from each cow for protein quantification, and the three cows with higher milk protein (average 3.81±0.54, >3.3%) were selected as the high milk protein group. In comparison, the remaining three cows (average 2.7±0.13, <3.0%) were chosen for the low milk protein group. Cows were housed at the farm, and consumption of alfalfa varieties was confirmed with the farm manager.

Whole blood and fresh milk samples were transported on ice to the lab and stored at −80°C until protein and nucleic acid extraction. Serum separation was conducted by collecting whole blood using 10ml conical centrifuge tubes without anticoagulant. After collection, samples were left overnight at 4°C, then centrifuged at 1,200×g at 4°C for 10 minutes. The supernatants were then transferred to new centrifuge tubes, centrifuged at 1,800×g at 4°C for 10 minutes, then collected and stored at −80°C.

Whey separation was conducted by collecting fresh milk samples that were centrifuged i) at 300×g at 4°C for 10 minutes, ii) at 3,000×g at 4°C for 20 minutes, and iii) at 10,000× g at 4°C for 30 minutes to remove i) fat, cells, and large debris, ii) milk proteins, and iii) other impurities, respectively. Supernatants were then collected and stored at −80°C.

### Sodium periodate oxidation test

A total of 10 mM sodium periodate was added to 10 μL of extracted bovine blood RNA sample, mixed, and oxidized at −20°C for 40 minutes. After centrifuging, 1 mL of absolute ethanol was added to resuspend the precipitate, which was incubated at −20°C overnight, and the RNA samples were centrifuged again at a low temperature. After absolute ethanol was discarded, 20 μL of enzyme-free water was added to dissolve the RNA precipitate, and the RNA in the samples was completely oxidized. The alfalfa-derived mtr-miR162, bovine-derived bta-miR-25, and bta-miR-16a were quantified in the oxidized RNA solution, and the plant-derived miRNAs were verified.

### RNA extraction and quantitative real-time polymerase chain reaction analysis

Total RNA was isolated from transfected bMECs in addition to bovine milk serum or whey using the RNAiso Plus/RNAiso Blood isolation kit according to the manufacturer’s instructions (Takara Biomedical Technology Co., Ltd., Beijing, China). The concentration, purity, and integrity of total RNAs were investigated using electrophoresis and Multi-Mode Reader (BioTek, Winooski, VA, USA). Complementary DNA (cDNA) was then synthesized using the PrimeScript RT reagent kit with the gDNA Eraser kit (Takara Biomedical Technology Co., Ltd, China) following the manufacturer’s protocol. Gene expression was assessed by quantitative polymerase chain reaction (qRT-PCR) using CFX-96 Touch Real-Time PCR instrument (BioRad, Hercules, CA, USA) real-time fluorescence quantification system and the ChamQ Universal SYBR qRT-PCR Master Mix real-time fluorescence quantitative PCR kit (Vazyme Biotech Co., Ltd, Nanjing, China). Primer Premier 5.0 (Premier Biosoft, San Francisco, CA, USA) software was used to design qRT-PCR primers for its related genes (Supplementary Table S1). The qRT-PCR reaction was carried out in a 20 μL PCR mix consisting of 10 μL of ChamQ SYBR qRT-PCR Master Mix, 0.4 μL of 100 μM gene primers, and 2.0 μL cDNA (100 ng/μL). The PCR reaction conditions were as follows: pre-denaturation 95°C for 30 seconds, 95°C for 10 seconds, and 51.7°C for 30 seconds throughout 44 cycles for CSN2; pre-denaturation 95°C for 30 seconds, 95°C for 5 seconds, and 60°C for 30 seconds for 40 cycles for other genes. The genes of glyceraldehyde 3-phosphate dehydrogenase (GAPDH) and 18S rRNA were employed as internal references, respectively.

### Cell culture

bMECs and the human kidney epithelial cells (HEK-293T) used in this study were provided by Prof. Wang Xingping’s research group in the Key Laboratory of Ruminant Molecular Cell Breeding in Ningxia Hui Autonomous Region [11]. All cells used in this study were taken from the third passage after resuscitation. The bMECs were subcultured using Dulbecco’s modification of eagle’s medium (DMEM)/F12 (Hyclone, Logan, UT, USA) cell media containing 10% fetal bovine serum (FBS) (BI, Beit Haemek, Israel), penicillin (100 IU/mL), streptomycin (100 μg/mL), hydrocortisone (5 μg/mL), insulin (5 μg/mL) and prolactin (20 ng/mL) at 37°C, 5% CO_2_ concentration and 100% saturated humidity. HEK-293T cells were cultured in high sugar DMEM (Hyclone, USA) with 5% FBS (BI, Israel) at 37°C, 5% CO_2_ modified atmosphere, and saturated humidity.

### Cell transfection

The sequence for the mtr-miR162 mimic was UCGAUAA ACCUCUGCAUCCAG. The sequences for si-GNG11 were sense (5′-3′): GCAAAGAAGUUAAGUUGCATT and antisense (5′-3′): UGCAACUUAACUUCUUUGCTT. miRNA mimic and a miRNA negative control (NC) were purchased from RiboBio Co., Ltd. (Guangzhou, China), si-GNG11 and si-NC were purchased from Sangon Biotech Co., Ltd. (Shanghai, China). Cells were transfected with 45 nM mtr-miR162 mimic, 45 nM NC, 80 nM si-GNG11, or 80 nM si-NC using a lipofectamine 3000 (Invitrogen, Carlsbad, CA, USA) transfection reagent for 48 hours at 37°C. Cells were used for studies after 48 hours of transfection.

### Cell counting kit-8 assays

bMECs were inoculated and cultured in 96-well plates. When cell density reached 60% to 70%, mtr-miR162 mimic, mimic NC, si-GNG11, or si-NC were transfected. After 48 hours, 10 μL of cell counting kit-8 (CCK-8) enhanced solution (Meilunbio, Dalian, China) was added, and the cell viability was calculated by reading at 450 nm absorbance light after 1 hour.

### 5-Ethynyl-2′-Deoxyuridine (EdU) Staining

bMECs were inoculated and cultured in 6-well plates. When cell density reached 60% to 70%, mtr-miR162 mimic, mimic NC, si-GNG11, or si-NC were transfected; after 48 hours, the proliferation of the bMECs was investigated using the BeyoClick EdU-555 Cell Proliferation Kit (Beyotime, Shanghai, China) according to the manufacturer’s protocol. Treated cells were observed and photographed with a fluorescence microscope (Olympus Corporation, Tokyo, Japan). The cytoplasms of newly proliferated cells were identified using red fluorescence, and the nuclei of all cells were identified using blue fluorescence.

### Cell apoptosis analysis

Cell apoptosis was detected via flow cytometry using the Annexin V-fluorescein isothiocyanate (FITC) apoptosis detection kit (Beyotime, China) according to the manufacturer’s protocols. Cells were digested with trypsin to detect apoptosis, and then a culture medium was added to stop digestion. Cells were collected in 1.5 mL centrifuge tubes spun at 1,000 g for 5 minutes, the supernatant was removed, and the pellet was resuspended in 1 mL phosphate-buffered saline. Next, cells were incubated in a water bath at 50°C for 2 minutes to stimulate apoptosis. Cells were collected via centrifugation and discarding the supernatant as before, and 195 μL Annexin V-FITC binding solution, 5 μL Annexin V-FITC, and 10 μL propidium iodide (PI) were added to the cell pellet. After incubation at room temperature for 20 minutes in the dark, cells were analyzed using flow cytometry.

### Enzyme-linked immunosorbent assay

Following transfection and subsequent incubation for 48 hours, the supernatants of the bMECs cultures from each treatment group were collected, and the secretion of α-casein, β-casein, and κ-casein was detected using the relevant enzyme-linked immunosorbent assay (ELISA) kits (CZi Bio CO., Ltd., Shanghai, China) according to the manufacturer’s instructions.

### Dual-luciferase reporter gene assays

The target mRNA of alfalfa xenomiR-162 was predicted using the TargetScan database (http://www.targetscan.org/vert_72/), and the presence of a targeting relationship was initially determined by RNAhybrid (https://bibiserv.cebitec.uni-bielefeld. de/rnahybrid/submission.html) comparison of the mature alfalfa xenomiR-162 sequence and the 3′ untranslated region (UTR) sequence of GNG11 using a dual-luciferase reporter gene assay. Wild-type (wt) and mutant (mut) GNG11 were cloned into the psiCHECKTM-2 plasmid using XhoI and NotI restriction sites. Then, 293T cell lines were cultured in 24-well plate culture dishes and transfected when 70% confluence was reached. Cells were transfected after 48 hours using the Dual-Luciferase Reporter Assay System (Promega, Madison, WI, USA) to determine the fluorescence intensity according to the manufacturer’s instructions.

### Western blot

The total protein of the bMECs was extracted with a whole protein extraction kit (KeyGEN, Nanjing, China), and its concentration was determined using a bicinchoninic acid protein assay kit according to the manufacturer’s instructions (KeyGEN, Jiangsu, China). A total of 100 μg protein extracts were loaded, then separated using 10% sodium dodecyl sulfate polyacrylamide gel electrophoresis and transferred onto polyvinylidene difluoride membranes. The extracts were blocked in a blocking solution at room temperature for 15 minutes, and the membranes were incubated with antibodies. Primary antibodies against GAPDH (AB0036, 1:3,000; Abways, Shanghai, China), proliferating cell nuclear antigen (PCNA) (D220014, 1:500; Sangon Biotech, China), and cyclin-dependent kinase2 (CDK2) (1:500; Sangon Biotech, China) were used, and the secondary antibody was goat anti-rabbit IgG (ZB-2301, 1:20,000; ZSGB-Bio, Beijing, China). A chemiluminescent ECL Western blot system (Tanon-5200; Tanon, Shanghai, China) was used for signal detection, and protein abundance was measured using Image J software.

## Data analysis

A minimum of three biological replicates and three technical replicates were performed for each experiment, and the relative expression was calculated using the 2–ΔΔcq formula. The test results were plotted and analyzed for significance by Student’s t-test using GraphPad Prism8 software (GraphPad Software, Inc., La Jolla, CA, USA), with p<0.05 as a significant difference (*), and p<0.01 as highly significant difference (**).

## RESULTS

### Alfalfa xenomiR-162 persists in bovine serum and milk and differentially expressed between the low milk protein group and high milk protein group

**Figure 1:**
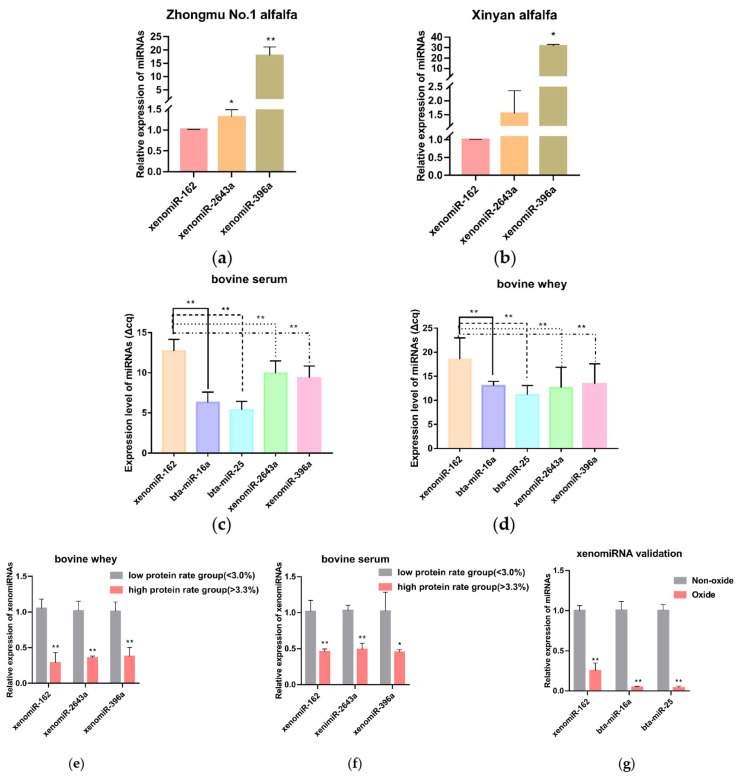
Alfalfa xenomiR-162 persists in bovine serum and milk. (a) qPCR detection of alfalfa miRNA levels in fresh samples of Zhongmu No. 1 alfalfa. (b) qPCR detection of alfalfa miRNA levels in fresh samples of Xinyan alfalfa. (c, d) Detection of endogenous and exogenous miRNAs in bovine whey and serum. (e, f) Detection of exogenous miRNAs in bovine serum and whey high protein and low protein groups. (g) Effects of sodium periodate oxidation on bovine and alfalfa miRNA transcript abundance. qPCR, quantitative polymerase chain reaction. * p<0.05, ** p<0.01.

According to the preliminary sequencing results of our research group [8], xeno-miR396a-5p, xeno-miR2643b-3p, xeno-miR2643a, and xenomiR-162 were the most highly differentially expressed in dried samples (Table 1). Their expression levels were validated by qRT-PCR (Figure 1a, 1b), which supported the reliability of the sequencing data and led us to focus on these four alfalfa miRNAs in subsequent experiments. Then, to investigate the expression and relation of xenomiRNAs with milk content, qRT-PCR was used to analyze the expression level of four alfalfa miRNAs and two cow miRNAs, which revealed that they were all detectable in bovine sera (Figure 1c), and in bovine whey (Figure 1d), with xenomiR-162 shows a highest expression level. Notably, a comparison of their levels in serum and whey from high and low milk protein groups revealed that the alfalfa xenomiR-162, xeno-miR2643a, and xeno-miR396a were differentially abundant in these milk protein groups (p<0.05), with xenomiR-162 accumulating to significantly higher levels in low protein whey and sera (Figure 1e, 1f).

The G+C contents of alfalfa xeno-miR396a-5p, xeno-miR2643b-3p, xeno-miR2643a, and xenomiR-162 were 38%, 33%, 33%, and 48%, respectively (Table 1). To further verify that xenomiR-162 indeed originated in plants, qRT-PCR was used to quantify the effects of sodium periodate on miRNA oxidation, and its transcript levels before and after oxidation were compared with that of bovine bta-miR-16a (27% G+C content) and bta-miR-25 (36% G+C content). This analysis showed that xenomiR-162 transcript abundance decreased by approximately 75% after oxidation, whereas bta-miR-16a and bta-miR-25 decreased by 95%, supporting the likelihood that xenomiR-162 detected in bovine milk or serum was produced in alfalfa, based on its relative resistance to sodium periodate oxidation (Figure 1g).

These results demonstrate that xenomiR-162 was highly expressed in alfalfa and was detected in bovine blood and whey, meanwhile, xenomiR-162 was differentially abundant in high and low milk protein groups, and it could resist the oxidation of sodium periodate. Thus, alfalfa-derived xenomiR-162 was screened for subsequent test validation.

### Alfalfa xenomiR-162 negatively affects bovine mammary epithelial cell proliferation and milk protein synthesis by altering mTOR pathway-related gene expression in bMECs

**Figure 2:**
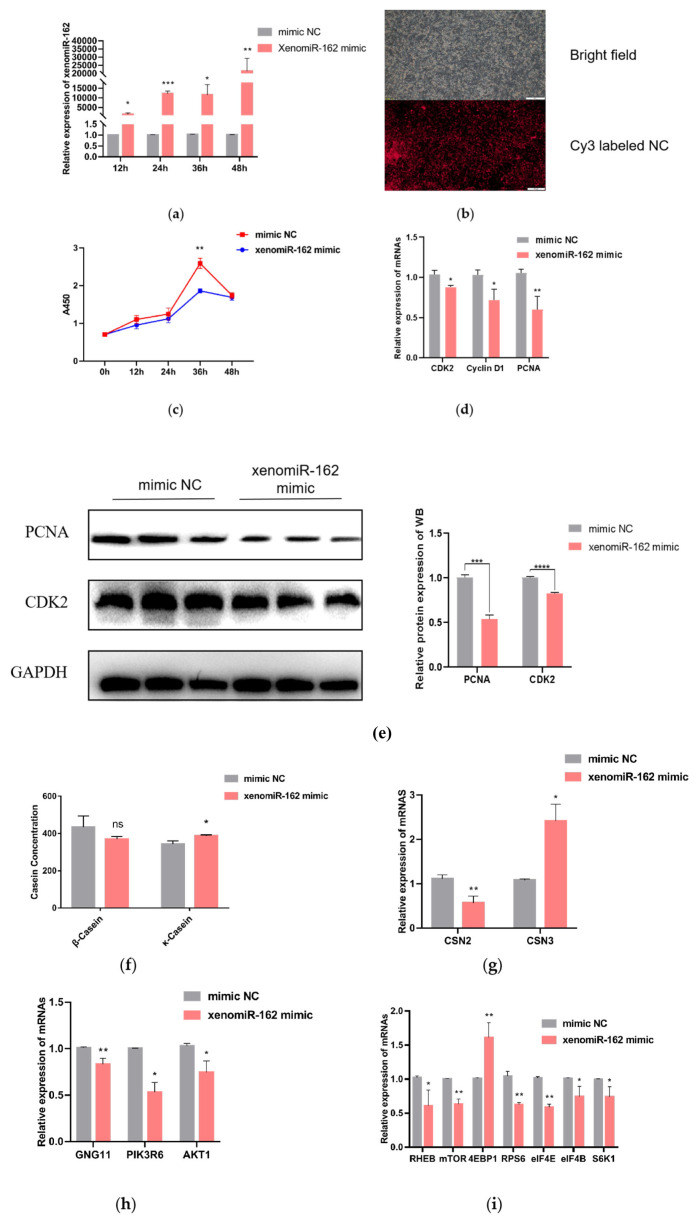
Effect of synthetic xenomiR-162 mimic proliferation by bMECs and milk protein synthesis-related genes after overexpression of xenomiR-162 in alfalfa. (a, b) The efficiency of alfalfa xenomiR-162 at 48 hours of transfection. Scale bar, 100 μm (c) CCK8 assay of bMECs proliferation at different time points over 2 days following transfection with synthetic duplex xenomiR-162 mimic. (d) Relative transcription levels of proliferation marker genes detected by qPCR. (e) Western Blot results of the proliferation-related proteins PCNA and CDK2. (f) The protein levels of casein β-casein and κ-casein were determined by ELISA. (g) Relative expression of genes encoding casein. (h) Relative transcription of GNG11 and its target genes with or without exposure to xenomiR-162 duplex mimic. (i) Expression levels of mTOR pathway genes involved in milk protein synthesis. The experimental results were analyzed by Student’s *t*-test. bMECs, bovine mammary epithelial cells; CCK8, cell counting kit-8; qPCR, quantitative polymerase chain reaction; PCNA, proliferating cell nuclear antigen; CDK2, Cyclin-dependent kinase2; ELISA, enzyme-linked immunosorbent assay; GNG11, G protein subunit gamma 11; mTOR, mammalian target of rapamycin. * p<0.05, ** p<0.01, *** p<0.001, **** p<0.0001.

To better understand the effects of xenomiR-162 accumulation in bovine serum and whey, a synthetic miR-162 mimic was used to transfect bMECs. qRT-PCR was used to determine the best transfection efficiency, and the result showed that the best transfection efficiency was 48 hours after transfection (Figure 2a). The transfected cells were observed for cy3-labeled mimic NC under an inverted fluorescence microscope and showed good transfection efficiency (Figure 2b). Then, CCK-8 and qRT-PCR were used to show the effect of xenomiR-162 on the proliferation of bMECs. The results of CCK-8 assays indicated that bMECs proliferation was indeed significantly inhibited under high levels of xenomiR-162 mimic at 36 hours after transfection (Figure 2c) (p<0.01), while qRT-PCR indicated that CDK2, Cyclin D1, and PCNA expression were also decreased at the mRNA level 48 hours after transfection (Figure 2d) (p<0.05). In addition, the effect of xenomiR-162 on the proliferation of bMECs was further investigated by measuring the expression levels of the proliferation-related proteins PCNA and CDK2, and the results showed the same difference as the mRNA level (Figure 2e).

To then confirm that accumulation of xenomiR-162 in dairy cows could ultimately affect casein synthesis, qRT-PCR analysis was used to quantify the expression casein-related genes (*CSN2* and *CSN3*) in the presence of high levels of the xenomiR-162 mimic, and the secretion levels of β-casein and κ-casein were detected using ELISA. The results revealed that CSN2 transcript levels decreased during the accumulation of xenomiR-162 (p<0.01), whereas the expression of CSN3 significantly increased (p<0.05) in bMECs (Figure 2g). ELISA results showed that over-expression of xenomiR-162 could reduce β-casein concentration but not significantly (p>0.05), while significantly it increased κ-casein concentration (Figure 2f) (p<0.05). This result may be because the spatial and temporal variations of mRNAs, as well as the local availability of resources for protein biosynthesis, strongly influence the link between protein levels and their coding transcripts.

After transfection of xenomiR-162 mimics for 48 hours in bMECs, qRT-PCR showed that GNG11, PIK3R6, and AKT1 were significantly downregulated compared with mimic NC (Figure 2h) (p<0.05). Based on these results showing that the expression of PIK3R6 and AKT1 was inhibited in the presence of the alfalfa xenomiR-162 mimic, most likely through interaction with GNG11, we next examined whether the duplex xenomiR-162 mimic could also affect the expression of genes related to milk protein synthesis that are known downstream targets of mTOR signaling, including Ras homolog, mTORC1 binding (RHEB), mTOR, ribosomal protein S6 (RPS6), eukaryotic translation initiation factor 4E (eIF4E), eIF4B, and ribosomal protein S6 kinase B1 (S6K1). Further relative expression analysis showed that all these genes were indeed significantly down-regulated in the presence of miR-162 mimic, but not the mimic NC (p<0.05), except for eukaryotic translation initiation factor 4E binding protein 1 (4EBP1), which was significantly up-regulated (Figure 2i) (p<0.01). These cumulative results indicated that alfalfa xenomiR-162 could inhibit milk protein synthesis by differentially regulating the transcription of mTOR pathway genes via suppression of GNG11.

### Alfalfa xenomiR-162 targets the *GNG11* gene

**Figure 3:**
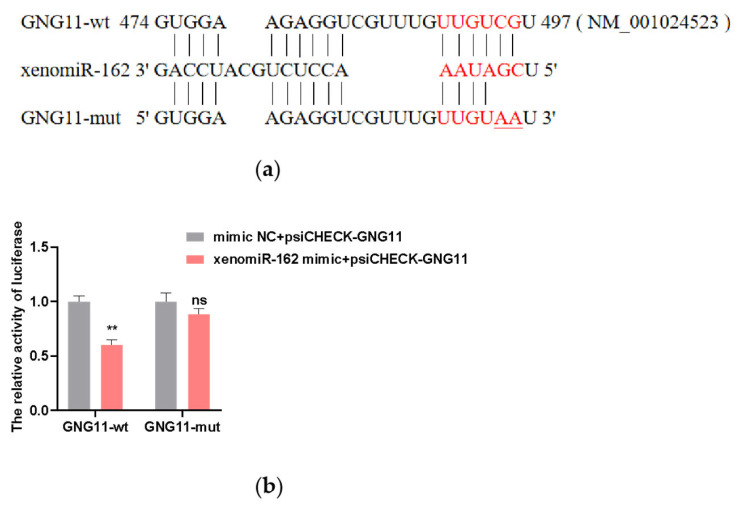
Alfalfa xenomiR-162 targeting of the GNG11 3′ untranslated region. (a) The binding site of alfalfa xenomiR-162 to GNG11. (b) dual-luciferase activity assays GNG11 in the presence of alfalfa xenomiR-162. The experimental results were analyzed by Student’s *t*-test. GNG11, G protein subunit gamma 11. * p<0.05, ** p<0.01.

To explore the possibility that xenomiR-162 may affect milk protein levels in dairy cows, we first sought to identify its possible regulatory targets in the bovine transcriptome. Prediction of xenomiR-162 targets using bioinformatic tools (TargetScan, miRanda, and RNAhybrid) revealed that it could bind to the 3′ UTR of the *GNG11* gene via complementary base pairing. To verify the interaction between xenomiR-162 and the 3′region of GNG11 regulation, we cloned the wild-type bovine GNG11 and generated a truncation variant harboring G→A and C→A conversions that could prevent base pairing with xenomiR-162 at the predicted 3′binding site (Figure 3a). The wild-type (WT) and truncated GNG11 constructs were each ligated into dual luciferase reporter plasmids and individually co-transfected into HEK293T cells with either synthetic duplex xenomiR-162 mimic or a non-targeted control RNA-mimic NC. The protein expressed by 293T cells has high repeatability and consistency among batches, and transfection with 293T cells can also eliminate the interference of some endogenous genes, thus, 293T cells were chosen for the dual luciferase test. After confirming the viability of transfected HEK 293T cells, detection of luciferase signal indicated that expression of WT GNG11-LUC in the presence of the xenomiR-162 mimic resulted in approximately 40% lower signal than that in cells transfected with mimic NC (p<0.01). By contrast, no significant differences in luciferase signal were observed between cells expressing the GNG11-mut-LUC and either xenomiR-162 or the mimic NC (p>0.05) (Figure 3b). Also, the expression level of GNG11 was significantly downregulated after overexpression of xenomiR-162 (p<0.01) (Figure 2h), suggesting that xenomiR-162 could specifically interact with the 3′ UTR of GNG11 and potentially interfere with its regulation.

### GNG11 silencing inhibited the proliferation of bMECs

**Figure 4:**
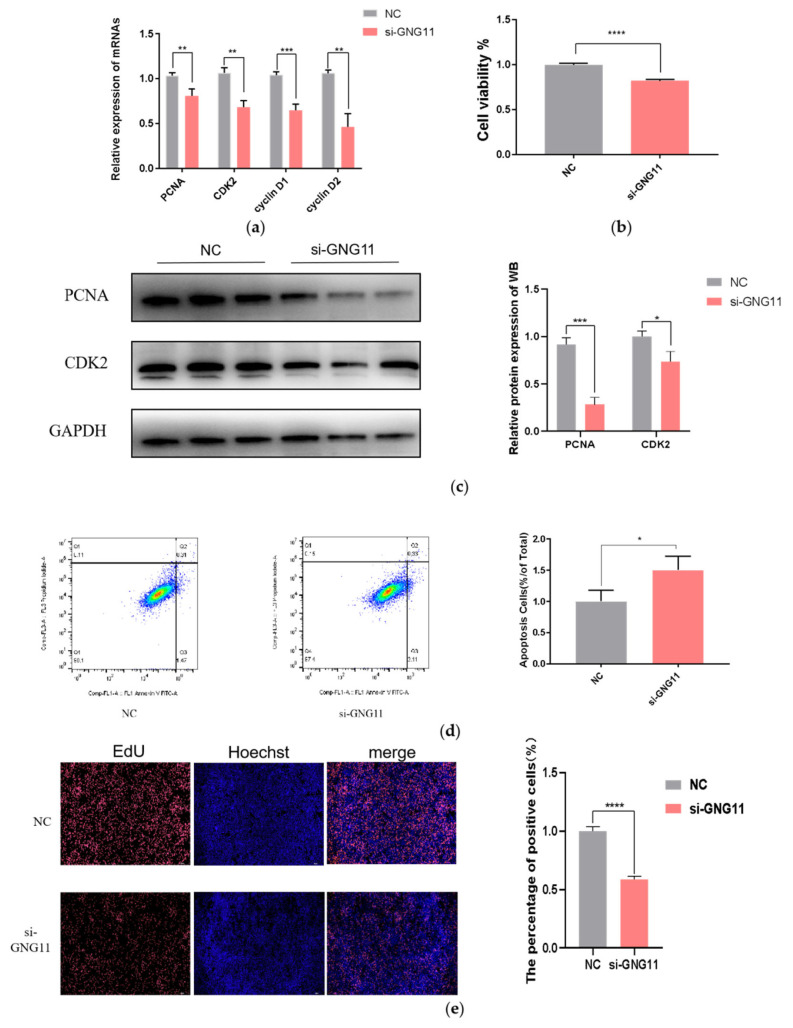
Effects of si-GNG11 on proliferation and apoptosis of bMECs. (a) The relative mRNA expression of proliferation marker genes. (b) CCK8 assay of bMECs proliferation at 48 hours with si-GNG11. (c) Western Blot results of the proliferation-related proteins PCNA and CDK2. (d) Flow cytometry results. (e) EdU results. Scale bar, 100 μm. The experimental results were analyzed by Student’s t-test. GNG11, G protein subunit gamma 11; bMECs, bovine mammary epithelial cells; CCK8, cell counting kit-8; PCNA, proliferating cell nuclear antigen; CDK2, Cyclin-dependent kinase2; Edu, 5-Ethynyl-2′-Deoxyuridine. * p<0.05, ** p<0.01, *** p<0.001, **** p<0.0001.

To study the effect of GNG11 on the proliferation of bMECs, si-NC, and si-GNG11 were transfected when bMECs reached about 70% confluence. The relative expression levels of proliferation marker genes (PCNA, CDK2, Cyclin D1, and Cyclin D2) were measured 48 h after si-GNG11 transfection by qRT-PCR. The results showed that the relative mRNA expressions of proliferation marker genes were significantly downregulated (Figure 4a) (p<0.01). In addition, the effect of GNG11 on the proliferation of bMECs was further investigated by measuring the expression levels of the proliferation-related proteins PCNA and CDK2, and the results showed the same difference as the mRNA level (Figure 4c). The CCK-8 results showed that cell viability decreased significantly after GNG11 48 h interference compared to the control group (Figure 4b) (p<0.0001). Flow cytometry results showed that disrupting GNG11 significantly promoted apoptosis (Figure 4d) (p<0.05); cell proliferation was measured using the EdU kit, and the results showed that disrupting GNG11 extremely significantly inhibited the proliferation of bMECs (Figure 4e) (p<0.0001).

### GNG11 silencing negatively affects milk protein synthesis by altering mTOR pathway-related gene expression in bMECs

**Figure 5:**
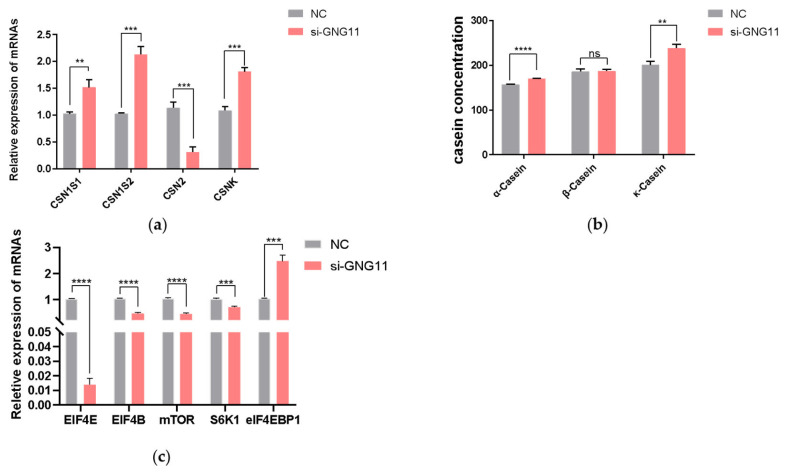
Effects of si-GNG11 on milk protein synthesis of bMECs. (a) The relative mRNA expression of casein encoding genes. (b) The protein levels of α-casein, β-casein, and κ-casein were determined by ELISA. (c) Expression levels of mTOR pathway genes involved in milk protein synthesis. The experimental results were analyzed by Student’s *t*-test. GNG11, G protein subunit gamma 11; bMECs, bovine mammary epithelial cells; ELISA, enzyme-linked immunosorbent assay; mTOR, mammalian target of rapamycin. * p<0.05, ** p<0.01, *** p<0.001, **** p<0.0001.

After interference with GNG11, the expressions of the casein-coding genes and genes related to the mTOR signaling pathway (EIF4E, EIF4B, mTOR, S6K1, and eIF4EBP1) were measured at the mRNA level using qRT-PCR. The results showed that the expression of CSN1S1, which encodes αS1-casein was significantly upregulated (p<0.01), *CSN1S2* gene (encoding αS2-casein), and *CSNK* gene (encoding κ-casein) were also significantly increased (p<0.001). In contrast, the *CSN2* gene (encoding β-casein) was significantly downregulated (Figure 5a) (p<0.001). To further test the content of casein in the supernatant of bMECs after disrupting GNG11, after transfection of si-NC and GNG11 interfering fragments into bMECs for 48 hours, α-casein, β-casein, and κ-casein concentration was measured using an ELISA detection kit. Results showed that compared with the control group, the α-casein and κ-casein level in the si-GNG11 experimental group was significantly higher than the control group (p<0.0001), and β-casein level was significantly lower than the control group (Figure 5b) (p>0.05), consistent with mRNA expression, indicating that GNG11 promotes the synthesis of α-casein and κ-casein and reduces β-casein synthesis in bMECs. The expression of EIF4E, EIF4B, mTOR, and *S6K1* genes were all significantly downregulated (p<0.001), while the expression of eIF4EBP1 showed the opposite trend (Figure 5c) (p<0.001). It is hypothesized that GNG11 may regulate the expression of *EIF4E*, *EIF4B*, *mTOR*, *S6K1*, and *eIF4EBP1* genes through the mTOR signaling pathway.

## DISCUSSION

The mammary epithelial cells of lactating cows can synthesize and divide lactating proteins, and the number of cells that secrete milk and their activity level greatly affect the ability of lactating cows to produce milk [12]. Therefore, it is essential to study the proliferation and activity of mammary epithelial cells to improve milk production. In this study, xeno-miRNAs were detected in the blood and milk of both high-protein-milk and low-protein-milk dairy cows, demonstrating that xeno-miRNAs could be absorbed into the tissues of dairy cows. This was consistent with previous studies that demonstrated direct uptake of plant miRNAs into animals without special treatment [13,14]. XenomiR-162 showed greater structure stability than the other three miRNAs due to the highest G+C content. Because the high G+C content of plant miRNAs could help them resist degradation by nucleic acid exonucleases in animals, stronger resistance to oxidation would efficiently distinguish plant miRNAs from animal miRNAs [15], this result was consistent with Zhang et al [3] and Li [16] observed that plant miRNAs remained essentially unchanged. In contrast, without any modification, most animal miRNAs like miR-483-5p, miR-16, and miR-221 standards were barely detectable after oxidation. Also, xenomiR-162 was differentially expressed in high and low-protein serum and whey. Based on these results above, we speculated that xenomiR-162 may have influenced the activity of genes that control milk protein metabolism in dairy cows.

This study found that alfalfa miR-162 has a cross-border regulation effect on bMECs, which could target the 3′ UTR of GNG11 to down-regulate its expression. GNG11 is a guanine nucleotide-binding protein (G protein) component that encodes a lipid-anchored cell membrane protein [17]. G proteins are composed of α, β, and γ subunits and play a crucial role in delivering extracellular stimuli to intracellular effectors. When ligand binds to G protein-coupled membrane receptor, G proteins dissociate into GTP-bound α and βγ subunits [18]. Current research on GNG11 is not comprehensive and mainly focuses on human diseases such as intestinal disease [19], lung cancer [20], bladder cancer [21], and breast cancer [22], with research on GNG11 in cows being underrepresented. The regulatory effects of GNG11 in dairy cows mainly focus on heat stress [23]. However, the impact of milk protein synthesis is not clear. Therefore, studying the effect of GNG11 on bMECs proliferation and milk protein synthesis can provide a theoretical basis for clarifying the regulatory role of the *GNG11* gene in breast tissue.

The proliferation and activity of bMECs are essential for improving milk production and content [24]. Over-expression of alfalfa xenomiR-162 and GNG11 silencing inhibited the cellular proliferation viability of bMECs. This is because the expression of cell-proliferation-related marker genes CDK2, Cyclin D1, Cyclin D2, and PCNA significantly reduced in the test group. The inhibiting effect was similar to the alfalfa miR-5754, specifically targeting MALAT1 [25]. Cyclin-dependent kinase 1, complexed with cyclin B, drives mitosis, while CDK2 drives S phase entry and replicon initiation [26]. Cyclin D1, associated with CDK4/6, acts as a mitogenic sensor, integrating extracellular mitogenic signals and cell cycle progression [27]. Proliferating cell nuclear antigen is highly expressed in proliferative cells. PCNA is involved in various cellular activities, including cell cycle control, DNA damage response, and chromatin assembly, and plays a central regulatory role in cell growth and development [28]. Cell proliferation runs throughout the entire lactation period of dairy cows. Therefore, it is crucial to study the proliferation of mammary epithelial cells in dairy cows for mammary development and lactation.

**Figure 6:**
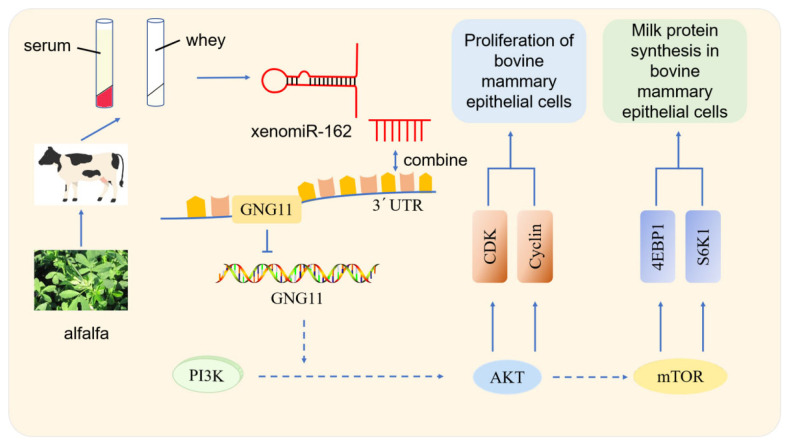
Summarized pathway of action of xenomiR-162 in bMECs. bMECs, bovine mammary epithelial cells.

Studies showed that some endogenous miRNAs play essential roles in the mTOR pathway regulating milk protein synthesis. Chen et al [29] observed that chi-miR-3031 activates the mTOR pathway and improves β-casein expression by downregulating insulin like growth factor binding protein 5 (IGFBP5). miR-574-5p inhibits mammalian targets of the PI3K/AKT-mTOR pathway by downregulating MAP3K9 and AKT3, leading to the presence of β-casein in goat GMECs [30]. In this study, over-expression of alfalfa xenomiR-162 and si-GNG11 significantly down-regulated the expression levels of PIK3R6, AKT1, mTOR, RPS6, eIF4E, RHEB, eIF4B, S6K1, and CSN2, while significantly up-regulated the expression of 4EBP1 and CSN3. PIK3R6 is the downstream gene of alfalfa xenomiR-162 that regulates GNG11. After overexpression of alfalfa xenomiR-162, GNG11 and PIK3R6 were down-regulated. Combined with dual-luciferase reporter gene assays, alfalfa xenomiR-162 regulated the mTOR signaling pathway by targeting GNG11 and affected milk protein synthesis. And ELISA results showed that over-expression of xenomiR-162 can decrease β-casein concentration but not significantly (p>0.05), while significantly increasing κ-casein concentration (Figure 2f) (p<0.05). κ-Casein is the only glycosylated casein consisting of galactose, sialic acid, and galactosamine. κ-casein cannot precipitate calcium cations because it consists of a low concentration of anionic phosphate groups; as a result, κ-casein provides stability to other proteins. However, this stability is eliminated upon rennet cleavage that forms para-κ-casein (hydrophobic) and caseinomacropeptide (CMP) (hydrophilic). CMP plays a crucial role in the overall digestion in individuals by augmenting serum gastrin secretion, decreasing gastric acid, and serotonin secretion, showing anticoagulant properties, minimizes platelet aggregation [31]. This indicates that alfalfa xenomiR-162 may target GNG11 and exert a regulatory role in milk protein synthesis, especially promoting the synthesis of κ-casein. Through the mTOR signaling pathway of dairy cows (Figure 6). It is worth noting that xenomiR-162 had the same effect of decreasing the relative expression of mTOR, as observed by alfalfa flavonoid supplementation [8].

Our study showed that xenomiR-162 could significantly up-regulate the expression of CSN3 despite its negative correlation with high-protein milk. However, we observed no direct conflict between low milk protein and high κ-casein at the mRNA level, since κ-casein is only one of the five types of casein proteins. In other words, although casein accounts for 80% of milk protein fractions, it does not reflect the whole protein content of milk proteins [32].

Furthermore, miRNAs have been known to be crucial epigenetic regulators that can respond to the environment, feed, and forage, regulate lactating-related key genes, affect the proliferation of bMECs, and, finally, affect the lactating function of mammary glands. For example, the bta-miR-29s family could inhibit DNA methylase DNA (cytosine-5-)-methyltransferase 3 alpha (DNMT3A) and DNMT3B (3 beta). This would decrease the methylation in lactating key genes and, ultimately, up-regulate the lactating function of bMECs [33]. An interesting question concerns if alfalfa xenomiR-162 and other xeno-miRNAs (with a particular 2′-O-methylated structure) would contact methylase DNMT3A and DNMT3B, and how would endogenous and xeno-miRNAs respond (by collaboration or completion) when both target the same genes. These and other questions warrant further research into the mechanism and function of xenomiRNAs.

## CONCLUSION

Alfalfa xenomiR-162 appears to regulate bMECs proliferation and milk protein synthesis via GNG11 in the mTOR pathway, suggesting that xenomiR-162 could be harnessed to modulate CSN3 expression in dairy cows and increase κ-casein contents in milk.

## Figures and Tables

**Table 1:** Characteristics and expression levels of alfalfa miRNAs analyzed in this study

miRNA	miRNA mature sequence (5′-3′)	Xinyan alfalfa (Transcripts per kilobase per million mapped reads, TPM)	Zhongmu No.1 alfalfa (TPM)	G+C content (%)
Xeno-miR396a-5p	UUCCACAGCUUUCUUGAACUU	105,374.70	94,603.75	38
Xeno-miR2643b-3p	UUUGGGAUCAGAAAUUAGAGA	10,161.60	6,975.89	33
Xeno-miR2643a	UUUGGGAUCAGAAAUUAGAGA	10,035.44	6,881.68	33
xenomiR-162	UCGAUAAACCUCUGCAUCCAG	6,165.89	3,154.56	48

## SUPPLEMENTARY MATERIAL

**Supplementary Table S1**. Information for qPCR
