# Supplementary material for: Alfalfa xenomiR-162 targets G protein subunit gamma 11 to regulate milk protein synthesis in bovine mammary epithelial cells
Source: Anim Biosci. 2024 Jan 20;37(3):509–21. doi: 10.5713/ab.23.0370 (PMC10915198; doi:10.5713/ab.23.0370)
Supplement: Supplementary file 1 [file ab-23-0370-Supplementary-Table-S1.pdf]

Table S1. Information for qPCR.

gene	sequence（5'-3'）	length of product（bp）
GNG11	F：GTTCTAGGGCGAAAATGCCG<br>R：AACGACCTCTTCCACTGAGG	277
PIK3R6	F：TGACAGCAGTGGAGCAGATG<br>R：TCCACAGGTGGAAGGTGATG	192
AKT1	F：GAGGAGATGGAGGTGTCGCT<br>R：GCTGTGGCCTTCTCCTTCAC	125
CSN2	F：GAGGAATCTATTACACGCATCA<br>R：TTTGTGGGAGGCTGTTAT	160
CSNK	F：GCCCAGGAGCAAAACCAAGA<br>R：GGACTTGGCAGGCACAGTAT	264
RHEB	F：ACAGCTGGGCAGGATGAATA<br>R：GACTCTGCTAACGCTTTCCCT	239
mTOR	F：AAAGGCATGTTCGAGGTGCT<br>R：GCTGCTTGGAGATTCGTCTG	211
4EBP1	F：CACTAGCCCTACAGGCGAT<br>R：GCTGGTGTCCACGAAGAAGA	298
RPS6	F：GAACATCTCTTTCCCGGCCA<br>R：AGGGGCTTTCGCACAACATA	477
eIF4E	F：AACGAGGAGGACGATGGCTA<br>R：AGCCGCTCTTAGTAGCTGTG	303
eIF4B	F：GTAGAAGAGCGGCTACAGA<br>R：GTTCCCGTTCCTGAGTTT	124
S6K1	F：CACCTGTTGACAGCCCAGAT<br>R：CGAGGGGATCGGATTTTTGG	139
GAPDH	F：GGCATCGTGGAGGGACTTATG<br>R：GCCAGTGAGCTTCCCGTTGAG	186
CDK2	F：ATGAACTGACCAGGAGGG<br>R：GCCAGGAGTTACTTCTATGC	115
Cyclin D1	F：CATGAACTACCTGGACCGCT<br>R：TCTTGGAGAGGAAGTGCTCG	260
Cyclin D2	F：CACCGATGTGGATTGCCTCA<br>R：TCCAGCTCATCCTCCGACTT	117
PCNA	F: TCCAGAACAAGAGTATAGC<br>R: TACAACAGCATCTCCAAT	94
mtr-miR-162	RT:GTCGTATCCAGTGCAGGGTCCGAGGT<br>ATTCGCACTGGATACGACCTGGAT<br>F: TCCGGTCGATAAACCTCTGC	55

	R: GGGTCCGAGGTATTCGCACT	
mtr-miR-2643a	RT: GTCGTATCCAGTGCAGGGTCCG<br>AGGTATTCGCACTGGATACGACTCTCTAAT<br>F: CGTTGATTTGGGATCAGAA<br>R: CAGTGCAGGGTCCGAGGTAT	63
mtr-miR-396a	RT:GTCGTATCCAGTGCAGGGTCCGAG<br>GTATTCGCACTGGATACGACAAGTTCAA<br>F: CGCGTTTTCCACAGCTTTC<br>R: AGTGCAGGGTCCGAGGTATT	62
bta-miR-16a	RT: CTCAACTGGTGTCGTGGAGTCGGCAA<br>TTCAGTTGAGCACCAA<br>F: GCCCGTAGCAGCACGTAAAT<br>R: TGTCGTGGAGTCGGCAAT	54
bta-miR-25	RT: CTCAACTGGTGTCGTGGAGTCGGCAAT<br>TCAGTTGAGTCAGAC<br>F: ACCCGCATTGCACTTGTCTC<br>R: TGTCGTGGAGTCGGCAAT	54
actin-7	F: GACCACCTACAACTCTATCA<br>R: AACCACCACTAAGAACAATG	80
18S	F: TTTCGATGGTAGTCGCTGTG<br>R: GGATGTGGTAGCCGTTTCT	98
